# Embryonic Diapause Is Conserved across Mammals

**DOI:** 10.1371/journal.pone.0033027

**Published:** 2012-03-12

**Authors:** Grazyna E. Ptak, Emanuela Tacconi, Marta Czernik, Paola Toschi, Jacek A. Modlinski, Pasqualino Loi

**Affiliations:** 1 Department of Comparative Biomedical Sciences, University of Teramo, Teramo, Italy; 2 Department of Experimental Embryology, Institute of Genetics and Animal Breeding of the Polish Academy of Sciences, Jastrzebiec, Poland; The Babraham Institute, United Kingdom

## Abstract

Embryonic diapause (ED) is a temporary arrest of embryo development and is characterized by delayed implantation in the uterus. ED occurs in blastocysts of less than 2% of mammalian species, including the mouse (*Mus musculus*). If ED were an evolutionarily conserved phenomenon, then it should be inducible in blastocysts of normally non-diapausing mammals, such as domestic species. To prove this hypothesis, we examined whether blastocysts from domestic sheep (*Ovis aries*) could enter into diapause following their transfer into mouse uteri in which diapause conditions were induced. Sheep blastocysts entered into diapause, as demonstrated by growth arrest, viability maintenance and their ED-specific pattern of gene expression. Seven days after transfer, diapausing ovine blastocysts were able to resume growth *in vitro* and, after transfer to surrogate ewe recipients, to develop into normal lambs. The finding that non-diapausing ovine embryos can enter into diapause implies that this phenomenon is phylogenetically conserved and not secondarily acquired by embryos of diapausing species. Our study questions the current model of independent evolution of ED in different mammalian orders.

## Introduction

Embryonic diapause (ED), or temporary arrest of embryo development, is a widespread phenomenon in the plant and animal kingdoms. ED is very “useful” in situations when further embryo development is risky, for example in harsh climates, in case of temperature or precipitation fluctuation, or due to maternally driven stimuli, such as during lactation [1]. In mammals, this protective phenomenon is maternally controlled [2] and can be experimentally induced in mice by ovariectomy, which prevents the ovarian estradiol surge that is necessary for implantation [3], [4]. ED occurrence is widespread in insects, fishes, birds and marsupials. Among placental mammals (*Eutheria*), it has been described in species that belong to all the highly represented orders (*Rodentia, Insectivora*, *Carnivora*, *Chiroptera*, *Edentata and Artiodactyla*), but not in *Primates*, in which the occurrence of this phenomenon was not explicitly investigated. In the largest mammalian order, *Rodentia*, ED was confirmed or surmised in a considerable number of species [1], [5]. In *Rodentia* and *Insectivora* the occurrence of post-partum oestrus that leads to pregnancy concomitantly with lactation may cause the induction of the so called ‘facultative’ ED. The other form of ED (‘obligate’ ED, because it occurs in all pregnancies) was described to be induced by environmental factors, for example photoperiod. In both forms, the physiological mechanism was recognized to be the same [6]. Only ‘facultative’ diapause was described in *Rodentia* and both forms in *Insectivora*. In other orders, the occurrence of an exclusively ‘obligate’ form was postulated [6]. Since ED is associated with unfavorable environmental conditions that may harm the developing embryo, ED could have been more widespread in the ancestors of currently living mammals, for example during glaciations [6], [7]. In particular, ‘facultative’ diapause was postulated to be a reminiscence of an ancestral strategy of mammalian development [5]. Nowadays, ED is less common in *Eutheria*, probably because this feature might be dispensable for species living in mild environmental conditions [8], [6]. Indeed, in ruminants (order: *Artiodactyla*) that live in a controlled, mild environment, ED has not been observed with the exception of the roe deer (*Capreolus capreolus*) [9], [10], [11], [12]. In this species, ED lasts 5 months and thus it extends the total period of pregnancy to 9 months, so that offspring are delivered in the optimal season, the spring. In the roe deer, the occurrence of ‘obligate’ ED is under photoperiodic control, a predictable factor, which is correlated with seasonal temperature variations and which generally precludes the survival of offspring that might have developed without the embryonic quiescence period. Nevertheless, instances of ED absence have been recorded in some cases [12].

Indeed, and conversely to what is generally believed, in several species characterized by ‘obligate’ diapause, its occurrence is actually flexible and variable in time [13], [14]. For example, the occurrence and the extent of ED in mustelids are correlated with their habitat, environmental temperature, litter size and female mating period [15], [16], [17]. In some cases, ED occurrence is restricted to subspecies that are geographically isolated [7]. ED can be also be induced in normally non-diapausing mustelids, such as the ferret (*Mustela furo*), by influencing their endocrine milieu [18]. Such flexible occurrence of ED suggests that the use of the term ‘obligate’ diapause may not be appropriate. Indeed, the optimal timing for reproduction may vary from year to year and from place to place. The flexible occurrence of ED in the same species supports the idea that ecological factors have a significant role in the determination of this trait [13], [14], [19]. This should be taken into account when classifying a species as diapausing or non-diapausing. The current knowledge about the variations of this trait in mammals is limited due to the difficulty of studying embryo development in wildlife. It is also rather complicated to establish to what extent ED occurs in mammals. Rodents are the most commonly used mammalian models for reproductive physiology studies and the flexible occurrence of ED was reported in lactating rats already 120 years ago [20]. Many routes might lead to induction of ED in a flexible fashion in rodents, such as pregnancy concomitant with heavy lactation, pre-puberty, elevated environmental temperature and even situations of social stress, such as overcrowding or presence of strange males [21], [22]. ED occurrence consequently to maternal stress has been hypothesized also in human concepti [23].

Since 1854 ED was known to occur in only one species belonging to *Artiodactyla*, the roe deer [9]. Much later, its occurrence was postulated also in other species belonging to this suborder (*Cervidae*), such as the Père David's deer (*Elaphurus davidianus*) [24], [1]. Also in other deer, gestation length was reported to be highly variable and influenced by various environmental factors [25]. Growing evidence of a much higher number of diapausing species (also in other orders) than previously estimated suggests that ED can be a basic reproductive phenomenon [26]. On the contrary, to date it is generally believed that ED *has evolved independently within different taxonomic groups of mammals* [27], [6], [7], [28]. Most probably due to this assumption, experiments on ED induction in mammals have not been carried out with the exception of one mustelid species, the ferret [29]. We propose that ED could be a fundamental, evolutionarily conserved phenomenon that is however not used in many species because no longer necessary. If this hypothesis is correct, embryonic diapause should be inducible in blastocysts from non-diapausing mammals, such as the domestic sheep, which is classified in the same suborder as the roe deer, but only the latter is recognized as a diapausing species. Here we show that diapause can be successfully induced in ovine blastocysts.

## Results

### Induction of embryonic arrest in blastocysts from a domestic species

**Figure 1:**
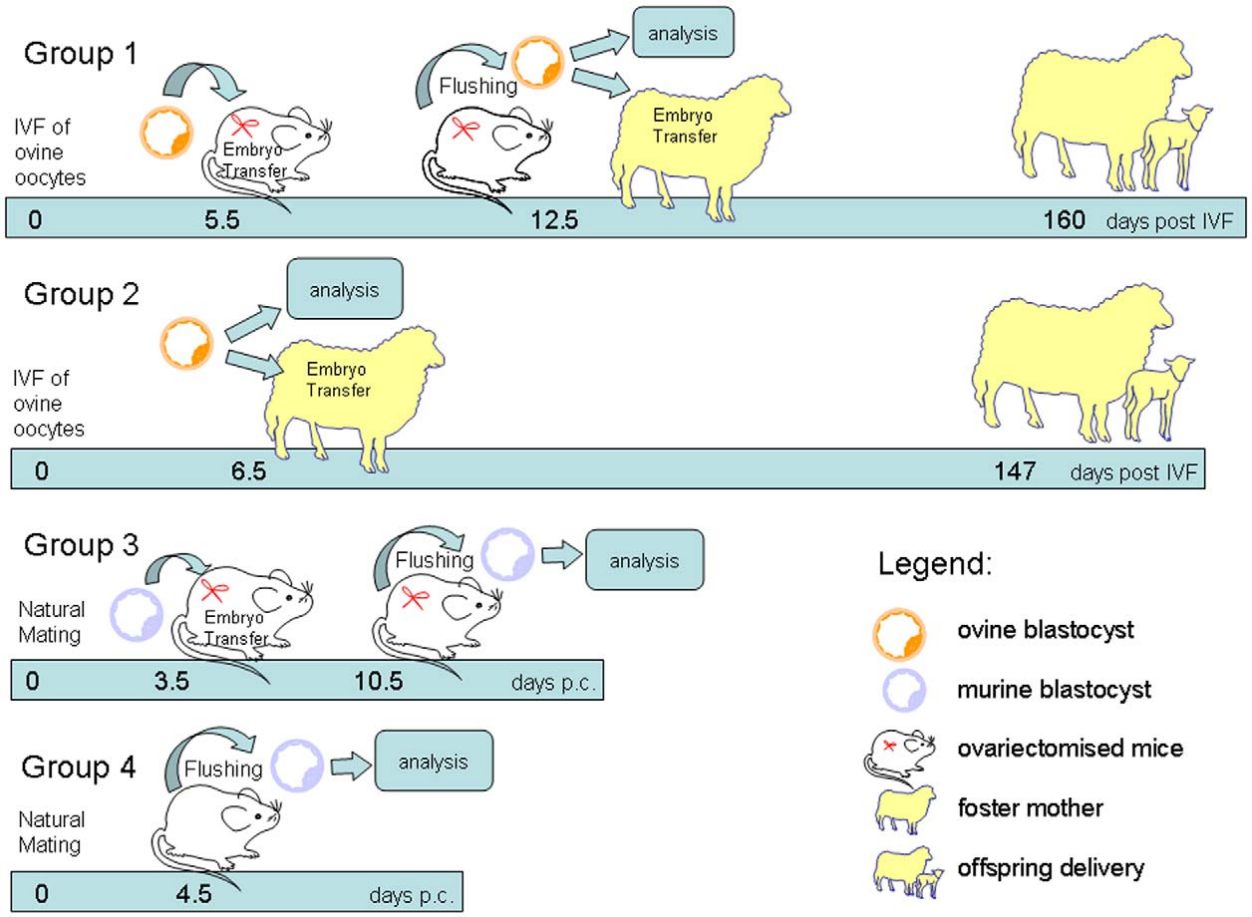
Experimental design of embryonic diapause (ED) induction in ovine blastocysts by transfer into ovariectomised pseudo-pregnant mice at 2.5 dpc. Following uterine flushing, diapausing ovine blastocysts were analyzed or transferred to foster ewes at day 6 after oestrus for full term development. The timing indicated in the diagram refers to embryos.

**Figure 2:**
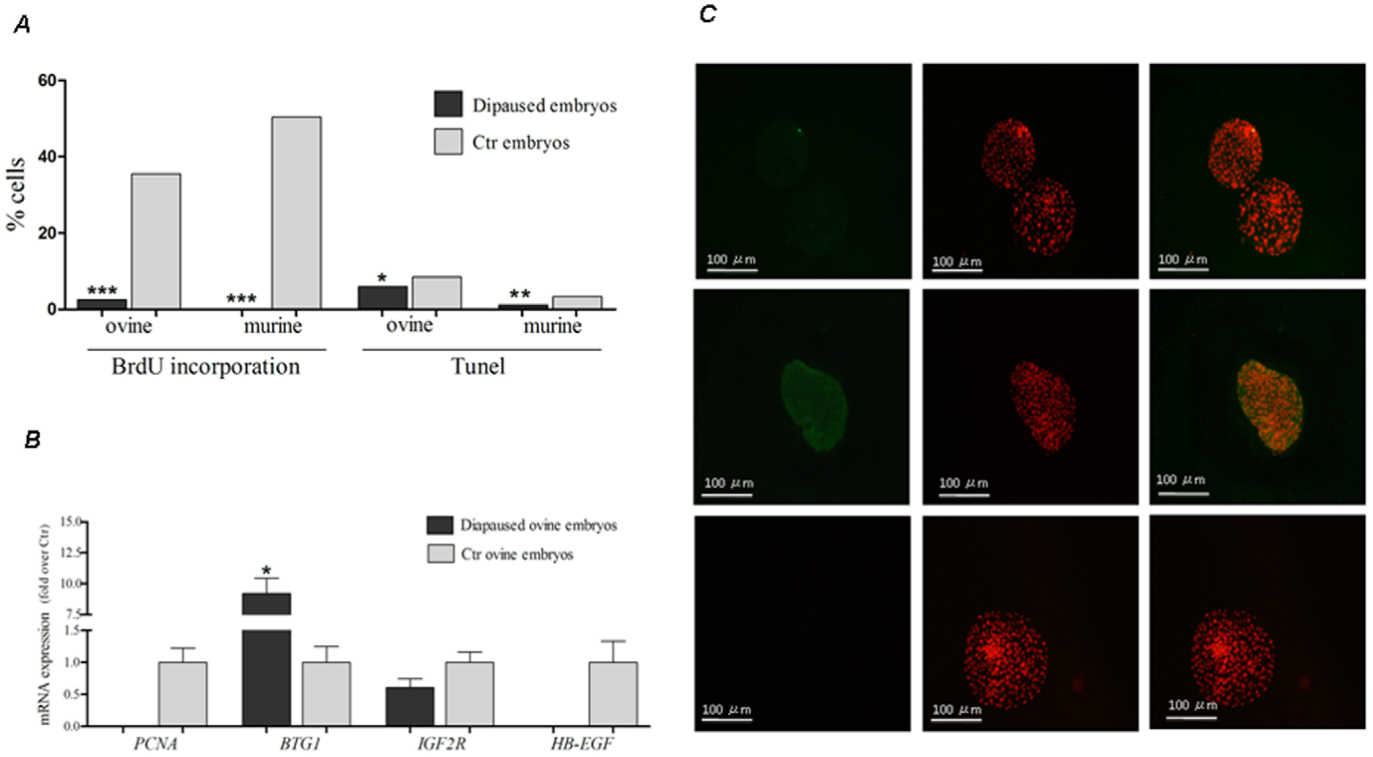
Confirmation of ED in ovine embryos. (*a*) Proportion of BrdU- and TUNEL-positive cells in diapausing ovine and murine blastocysts flushed from ovariectomised mouse uteri and in controls (*b*) qRT-PCR analysis of genes involved in ED control. Genes that positively regulate cell proliferation (*PCNA*) and signaling (*HB-EGF*) were not expressed in diapausing ovine blastocysts, while the anti-proliferative gene *BTG1* was significantly over-expressed. *IGF2R* mRNA expression did not differ statistically between diapausing and control blastocysts. (c) Immunolocalization of CB1 (green) in diapausing (middle panel) and control ovine blastocysts (upper panel). Nuclei (red) were visualized with propidium iodide. CB1 expression is higher in diapausing ovine blastocysts. Lower panel: ovine blastocysts incubated with neutralized anti-CB1 antibody showing no positive signal. For each experiment ≥5 blastocysts were used and it was repeated 3–5 times. Results are mean ± S.E.M. *** p<0.0001, ** p<0.003, *p<0.03.

To investigate whether ED could be induced in a domestic species, 856 early ovine blastocysts (Group 1), 150 ovine blastocysts (Group 2), 72 early mouse blastocysts (Group 3) and 27 mouse blastocysts (Group 4) were used (Figure 1). Seven days after transfer of early ovine (Group 1) and mouse blastocysts (Group 3) into uteri of pseudo-pregnant mice, in which diapause conditions were induced, uterine flushing was performed. From the inter-species (sheep-mouse) transfers (Group 1), 180/856 (21%) viable blastocysts were recovered as indicated by their re-expansion in culture within 1–2 hours. None of embryos had elongated, as normally observed in ruminants, and their size, according to the diameter of their *zona pellucida*, had not changed during the seven days. From intra-species (mouse-mouse) embryo transfers (Group 3), 35/72 (49%) of viable blastocysts were recovered. Diapausing, viable ovine and mouse embryos showed arrested DNA replication (assessed by BrdU incorporation), while both negative controls, represented by ovine blastocysts cultured *in vitro* (Group 2) and murine blastocysts flushed from intact mice (5.4 blastocysts/female) (Group 4), exhibited high level of DNA synthesis (Figure 2a). The percentage of dead cells was lower in diapausing ovine (P<0.018) and mouse (P<0.003) blastocysts than in controls. Moreover, mouse blastocysts showed a more marked decrease in the proliferation and cell death rate than ovine embryos (P<0.0001). Further analysis revealed differential expression of ED markers between diapausing and active ovine blastocysts (Figure 2b). Genes that positively regulate cell proliferation (*PCNA; Proliferating Cell Nuclear Antigen*) and signaling (*HB-EGF; Heparin-binding EGF-like growth factor*) were down-regulated, whereas the anti-proliferative *BTG1(B-cell Translocation Gene 1)* gene was strongly up-regulated. Conversely, the expression of *IGF2R* (*Insulin-like Growth Factor 2 Receptor*) did not differ significantly between diapausing and active ovine blastocysts. CB1 (*cannabinoid receptor type 1*), which is normally down-regulated before implantation, was highly expressed in diapausing ovine blastocysts (Figure 2c).

### Diapausing sheep embryos can develop to term

**Figure 3:**
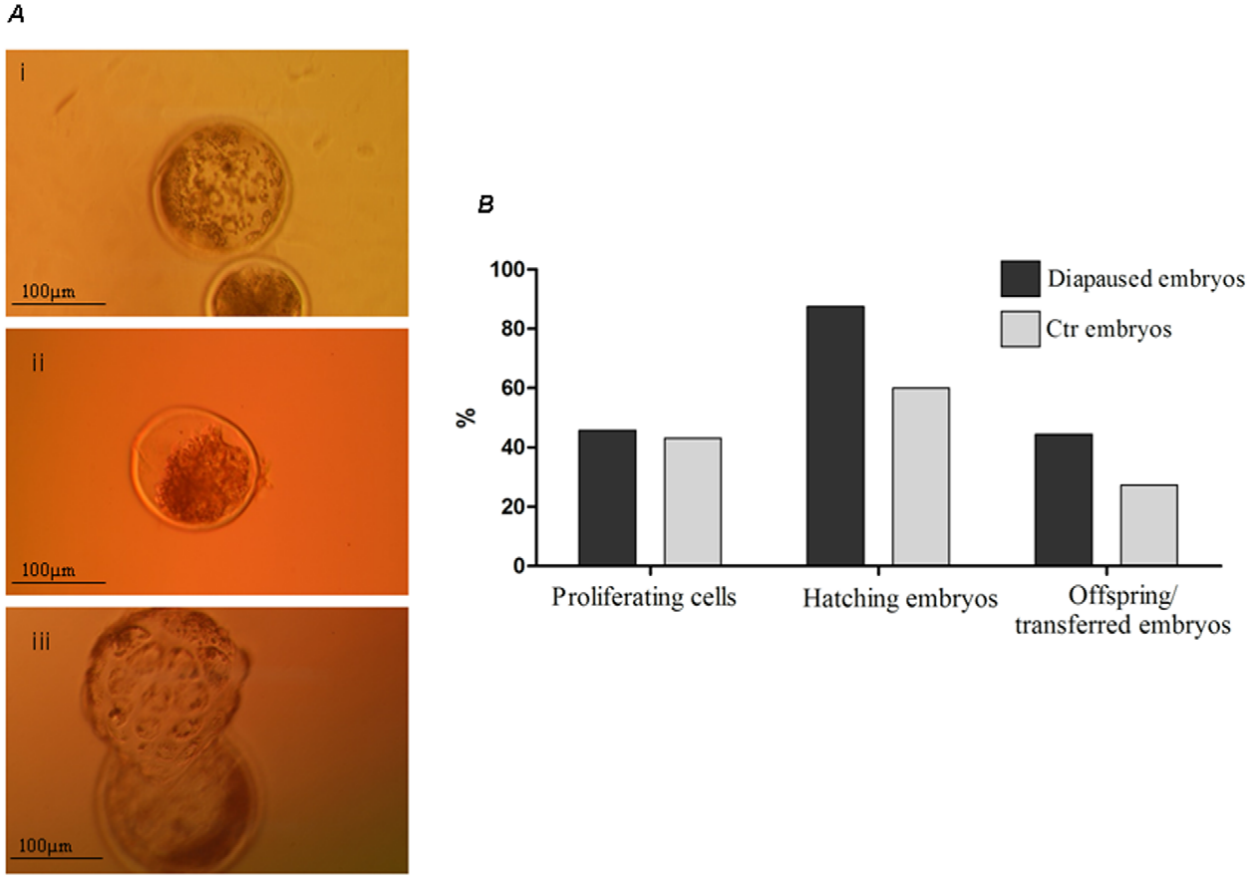
Reversibility of growth arrest in ovine embryos following flushing from the uterus of ovariectomised mice . (*a*) Ovine blastocysts before (i) and immediately after (ii) transfer to mouse uteri, and following 12 hours of culture in vitro (iii). (*b*) Percentage of BrdU-positive, proliferating cells and of embryos hatching from the *zona pellucida* in diapaused ovine blastocysts after 48 hours in culture and number of offspring developed from diapaused ovine blastocysts following their transfer into receptive uteri of foster ewes. Controls were in vitro cultured ovine blastocysts (day 6.5). For each experiment ≥5 blastocysts were used and it was repeated 3–5 times. Results are mean ± S.E. M *p<0.05.

The induction of diapause in ovine blastocysts was fully reversible (Figure 3). Diapausing ovine embryos restarted growing *in vitro* even at higher rate than control ovine blastocysts (Group 2). Furthermore, blastocysts hatched from their *zona pellucida* and after transfer to recipient sheep they developed to term (8 lambs/18 transferred blastocysts) at a proportion statistically comparable to control embryos (6 lambs/22 transferred blastocysts). The pregnancy length following transfer of diapausing blastocysts was similar to controls (147.4 vs. 148.1 days, respectively). All offspring had normal birth weight (2.5–3.9 kg) and were healthy.

## Discussion

These results show that embryos from a domestic mammal, the sheep, can enter into diapause when adequate conditions are created.

Mammalian embryos can develop independently until the blastocyst stage and then they recognize uterine signals necessary for their further development. If such signals are not sent by the uterus, embryos will stop or retard their growth. ED can therefore be explained as an adaptation of the embryo to environmental constraints. In our study, DNA synthesis, albeit at an extremely low level, was still observed in diapausing ovine blastocysts 7 days after transfer into pseudo-pregnant mice in which diapause conditions were induced. It is possible that blastocysts from sheep, a species which belong to *Artiodactyla*, grow slowly but continuously throughout the duration of diapause, as it occurs in its diapausing relative, the roe deer [11]. Alternatively, sheep blastocysts might need more time to completely stop proliferation. According to the works by MacLaren [30] and Copp [31], during the first days of diapause, murine blastocysts similarly slow down growth, while only around 7 dpc they are completely arrested. In agreement with these reports, no DNA synthesis was observed in diapausing mouse embryos in our study. Absence of DNA synthesis was also noted in diapausing rat, mink and fur seal embryos [32]. The down-regulation of *PCNA*, a cell cycle regulatory gene, also was observed in very distinct *taxa*: insects, mouse [33], [34] and sheep (present study). Also genes that regulate negatively cell proliferation, such as *BTG1*, are maximally expressed during ED both in mouse [34] and sheep. On the other hand, in contrast to what was reported in the mouse [34], the expression of *IGF2R*, which retards cell proliferation when over-expressed, did not differ between diapaused and active ovine blastocysts. Different regulation of *IGF2R* in ovine and murine diapaused embryos may be related to the different stage of ED in which the analysis were performed (2.0–2.5 days of ED vs. 5–6 days, respectively). It was demonstrated in the mouse that embryos entering a quiescent state convert only slightly (1%) their global gene expression pattern [34]. *HB-EGF* expression, the earliest indicator of embryo signaling to the uterus [35], [36], is down-regulated both in diapausing sheep (our study) and mouse blastocysts [34]. In active blastocysts, the Cannabinoid Receptor 1 (CB1) is constantly down-regulated, while high level of CB1 expression is observed during embryonic diapause [37]. CB1 is functional in mouse and ovine embryos [37], [38] and the present finding shows that CB1 down-regulation during ED is conserved in both species. Collectively, the molecular control of ED appears similar in very different animal species. Unfortunately, reports about molecular regulation of ED are not available for other mammalian species, probably due to the low interest in the subject and the experimental difficulties (low availability of gene sequences and antibodies).

It is fascinating that growth arrest in diapausing embryos does not lead to death. Even embryos which cannot implant (by exposing the female to constant darkness or following ovariectomy) are able to survive for long periods (up to 300 days in mustelids) [17]. In our study, cell death in both mouse and sheep diapaused blastocysts was even lower than in controls. An earlier study in which the cell death index in diapausing and active mouse embryos was compared did not reveal any differences in this value, although the ED duration was not the same [31]. Higher cell death in control ovine blastocysts may be attributed to the sub-optimal *in vitro* conditions used to obtain this group of embryos. However, a higher rate of cell death was also observed in control mouse blastocysts developed *in vivo*. Since programmed cell death regulates epiblast differentiation in actively growing blastocysts [39], [40], the decreased level of cell death in dormant blastocysts should not be surprising. It has been recently suggested that cell death is avoided during ED as there is no risk of oxidative stress because opening of the mitochondrial permeability transition pore and release of Cytochrome c do not occur [41]. Lower cell death during ED could be also due to DNA repairing activity [42].

With the exception of studies in the ferret (*Mustela furo*) [29], [18], there is no available scientific data about the experimental induction of ED in non-diapausing species. In ferret blastocysts, the *zona pellucida* was retained during ED [17]. Conversely, hatched blastocysts are observed during ED both in the mouse and roe deer, the only diapausing ruminant [11], [1]. This is because the *zona pellucida* is retained in blastocysts of diapausing species with inactive *corpora lutea*, such as mustelids or kangaroos, and not in species with active *corpora lutea*, such as roe deer. The *zona pellucida* provides physic-mechanical support for the embryos. Its retention may also provide protection from the uterine immune response. *Zona pellucida* was retained in diapausing ovine embryos in our experiments, which may be indicative of its immune-protective function in a situation in which embryos are placed in the uterus of another species. On the other hand, it is worth noting that immediately following flushing, broken *zonae* were observed in some diapaused ovine blastocysts. It cannot be excluded that hatched blastocysts were lost during flushing due to their fragility. Indeed, we and others [22] remarked that diapaused murine blastocysts are very fragile and they easily collapse during flushing. The two-fold lower recovery rate of diapausing ovine blastocysts in comparison to mouse blastocysts may be due to the ongoing immune attack against foreign embryos present in the uterus. In support to this suggestion, our preliminary control experiment (data not shown) demonstrated no embryos or sign of implantation in uteri of non-ovariectomised, pseudo-pregnant mice (at day 9.5 p.c.) in which ovine blastocysts were transferred for 7 days.

The phenotype of any diapausing embryo, also the ones described in this work, is similar among the mammalian species studied. First, *no physiological differences* of ED are observed among embryos of different orders of mammals. Second, the dormancy state is entered at *the same embryonic stage, the blastocyst*, in all mammalian species, notwithstanding the species-related differences in the subsequent development. For example, in mouse, dormant blastocysts will directly implant after activation, while in roe deer, blastocysts will necessitate a further week of development before implantation. Third, we show *the lack of species-specificity of the uterine conditions responsible for ED* as they can delay the development of naturally non-diapausing ovine embryos. The lack of species-specificity in the uterine conditions that regulate ED and implantation was previously demonstrated by inter-specific transfer of two diapausing rodents: rats and mice [43]. It was also demonstrated in *Carnivora*, in a work in which ferret embryos entered into diapause (although they normally do not) after transfer into the uterus of the mink (*Mustela vison*), a closely related diapausing mustelid [29]. Our study demonstrates that the uterus of *Rodentia*, in which ED is known to be ‘facultative’, can induced diapause in embryos of *Artiodactyla*, in which ED, when present, is classified as ‘obligate’. This finding supports the idea that similar mechanisms are involved in both ‘facultative’ and ‘obligate’ ED.

In mammals, the embryo-maternal cross-talk starts at the blastocyst stage. The metabolic activity is sharply reduced in developed blastocysts in comparison to earlier stage embryos [44], and there is essentially no need for maternal nutrition. Therefore, this stage represents the ideal moment for developmental arrest if the environmental conditions (including the maternal energy reserves) are inadequate for straightforward progression of pregnancy. The lack of maternal signaling has been suggested to be a cause of diapause entry [45]. Such view is supported by our experiments and also by other works in mice, where surgical removal of the ovaries precludes the occurrence of the estradiol surge, necessary for uterine receptivity [3]. Indeed, a maternal trigger is necessary to release the blastocyst from diapause and for its progression through development rather than to halt embryo development, as it occurs in birds, where the embryo proceeds to the blastodisc stage and arrests its development until incubation time (i.e. maternal signal). Indeed, embryonic dormancy and inability to develop further are related to the absence of the uterine signal for implantation. Such deficiency of embryo-uterine cross-talk takes place when mouse blastocysts are in a uterus in which LIF (which signals uterine receptivity) is not expressed in endometrium [46], [47]. As a consequence, embryos enter into diapause, but they maintain the ability to develop to term once transferred to wild type, pseudo-pregnant mice. Interestingly, the only report about ovine embryos that were rendered unable to implant concerns blastocysts developed in the uterus of females lacking the endometrial glands [48]. These embryos hatched and remained viable, but could not grow and elongate. Although they were not transferred to recipients in order to check whether they could be activated and develop further, our experiment demonstrates that diapausing ovine embryos are fully able to restart and proceed normally through development once placed in the receptive uterus of synchronized ewes.

Plausibly, ED is conserved in mammalian embryos and the conditions necessary to induce this state by non-diapausing females may not be difficult to put in place. It would be interesting to check if “older” embryos asynchronously transferred to “younger” uterus enter into diapause. The development of “older” embryos placed in “younger” uterus is retarded (rat: [49]; sheep: [50]; rabbit: [51]; horse: [52]). Notwithstanding of this retard, they develop more successfully than their synchronized counterparts, as demonstrated in mouse [53]; [54] and also in non-diapausing species (pig: [55]; rabbit: [56]). Other developmental benefits of the diapause for murine embryos includethe DNA repair of lethally irradiated embryos [42] and an extended survival of parthenogenetic embryos [57]. Furthermore, ED is considered to be a permissive state for embryonic stem cell derivation [58], [59]. Our findings might be of relevance for the development of alternative strategies for the isolation of embryonic stem cells from large animals. Inner cells mass can be isolated from diapausing sheep embryos and cultured in conditions favoring the maintenance of pluripotency, although different from mouse developmental stage and ensuing cells signaling should be taken into account. In mouse, the presence of LIF in culture is indispensable to derive embryonic stem cells (ESC) from undifferentiated inner cell mass. However, unlike in mouse, diapause entry in sheep occurs at more developmentally advanced blastocyst, in which the epiblast is already formed (just before gastrulation). Molecular signaling which trigger stem cells derivation and maintenance vary depending on the developmental stage of the embryo. Mouse embryonic stem cells may be also successfully derived using late epiblast [60]. For those cells, activin/Nodal pathway appears indispensable. Epiblast-derived pluripotent cell lines (EpiSC) from various species, such as mouse, rat or human are all functionally similar and independent on LIF/GP130 signaling [60], [61]. The establishment of embryonic stem cells from diapausing sheep blastocyst using chemically defined activin containing culture medium could be more advantageous than so far used unsuccessful approaches. Regardless of success of such strategies, basic studies on epi/genetic mechanism ensuring a better survival of the embryo entering diapause is highly needed.

The induction of ED in non-diapausing ovine embryos questions the current model about the independent evolution of diapause in different mammalian orders. This study provides a starting point to verify the flexible occurrence of ED in mammals and opens new perspectives for reproductive [62]; and evolutionary biology.

## Materials and Methods

### (a) Animals and embryos

Animal experiments were performed in accordance with the Italian Animal Protection Regulations (DPR 27/1/1992) and the Polish Government Act for Animal Care, in conformity with the European Community regulation 86/609. Animal experiments were performed within the permission of the Director of the Institute of Genetics and Animal Breeding, Polish Academy of Sciences to conduct experiments on mouse and sheep valid from 18.12.2009 till 30.12.2012 (permit number 4/2009). Swiss albino mice were kept in a temperature-controlled room with a 12 h light/dark cycle. A total of 24 female mice were mated with fertile males to obtain embryos while 120 females were mated with vasectomised males. Sheep blastocyst were produced *in vitro* as previously described [63], [38] and used for transfer into the uteri of pseudo-pregnant mice, in which diapause conditions were induced by ovariectomy and progesterone treatment [64], [65] (Group 1) or as negative control (Group 2) (Figure 1). As positive control (Group 3), early mouse blastocysts (3.5 days post-coitum - dpc) were similarly transferred into pseudo-pregnant ovariectomised mice. Embryos from both species were transferred to the uterus of ovariectomised mice (8 blastocysts/female) at day 2.5 of pseudo-pregnancy. After 7 days, embryos were flushed from mouse uteri and analyzed or transferred into recipient ewes. Mouse embryos flushed from the uteri of pregnant (non-ovariectomised) mice at 4.5 dpc served as negative control for mouse model (Group 4). Since diapausing embryos removed from the uterus resume activity and growth when placed *in vitro* or in a receptive uterus [3], [64], we also analyzed the ability of diapausing ovine blastocysts to restart development *in vitro* or after surgical transfer (in pairs) into 20 synchronized Sarda sheep recipients for full term development as described previously [63].

### (b) Embryo analysis

- **Immunofluorecence.** TUNEL (Terminal Deoxynucleotidyl Transferase-mediated dUTP nick-end labelling), CB1 immunodetection and thymidine analogue, 5-bromo-deoxyuridine (BrdU) incorporation in cells undergoing DNA synthesis was performed as we describe previously [38]. Additionally, to estimate the rate of BrdU incorporation in diapausing ovine and mouse blastocysts *in vivo*, ovariectomised (9.0 dpc) and control pregnant (4.0 dpc) mice received a single i.p. injection of 5 mg/ml BrdU dissolved in 0.9% NaCl with 0.007 M NaOH, at a dosage of 50 mg per kilogram of body weight. After 10 hours embryos were flushed from the uteri.
- *Expression analysis:* Expression analysis: Poly(A)+ RNA was isolated from single, frozen (PBS and 0.4% PVP) embryos using the Dynabeads mRNA DIRECT Kit (Invitrogen Dynal AS, Oslo, Norway) following the manufacturer's instructions. Reverse transcription was carried out using QuantiTect Reverse Transcription Kit (Qiagen, Milan, Italy). Obtained cDNA was used for Real-Time PCR to quantify the expression of HB-EGF, PCNA, IGF2R and BTG1 with the following primer pairs: HB-EGF (NM_001144090): *aatctggacacccctactca* and *cttgcctttcttctttcttttc* ; PCNA (NM_001034494): *aaggatctcatcaacgagg* and *tactagtgccaacgtgtcc* ; IGF2R (AF353513): *attacctgcaaagccagagc* and *ttgacttgagtggaatctgc* ; BTG1 (NM_001142887.1) *ctaagttcctccgcaccaag* and *cctcgccaattctgtaggac* . Amplification was performed using the Platinum SYBR Green qPCR SuperMix UDG with ROX (Invitrogen, Milan, Italy) and the ABI PRISM 7900 Real-time PCR System (Applied Biosystems, Carlsbad, USA) according to the manufacturer's instructions. To avoid false-positive signals, dissociation-curve analyses were performed at the end of each run. Relative gene expression values were calculated using the comparative threshold cycle (ΔΔCt) method with ß-Actin (NM_001009784) as endogenous control (primer pairs: *aatcgtccgtgacatcaagg* and *ttcatgatggaattgaagg* ).

### (c) Statistical analysis

The relative mRNA expression values were the mean (±SEM) of at least five independent determinations, each in triplicate. Statistical analysis was performed with the non-parametric Mann-Whitney T test (GraphPAD Software for Science, San Diego, USA). Differences were considered significant when P<0.05. Physiological parameters were reported as percentage of positive cells (or embryos) per total and analyzed using the Chi square test (GraphPAD Software for Science, San Diego, USA). The intensity of the fluorescent signal was measured using the confocal microscope LaserSharp 2000 and LaserPix software (Biorad, Milan, Italy).
